# Supplementary material for: The Reprimo-Like Gene Is an Epigenetic-Mediated Tumor Suppressor and a Candidate Biomarker for the Non-Invasive Detection of Gastric Cancer
Source: Int J Mol Sci. 2020 Dec 12;21(24):9472. doi: 10.3390/ijms21249472 (PMC7763358; doi:10.3390/ijms21249472)
Supplement: Supplementary file 1 [file ijms-21-09472-s001.pdf]

SUPPLEMENTARY MATERIAL

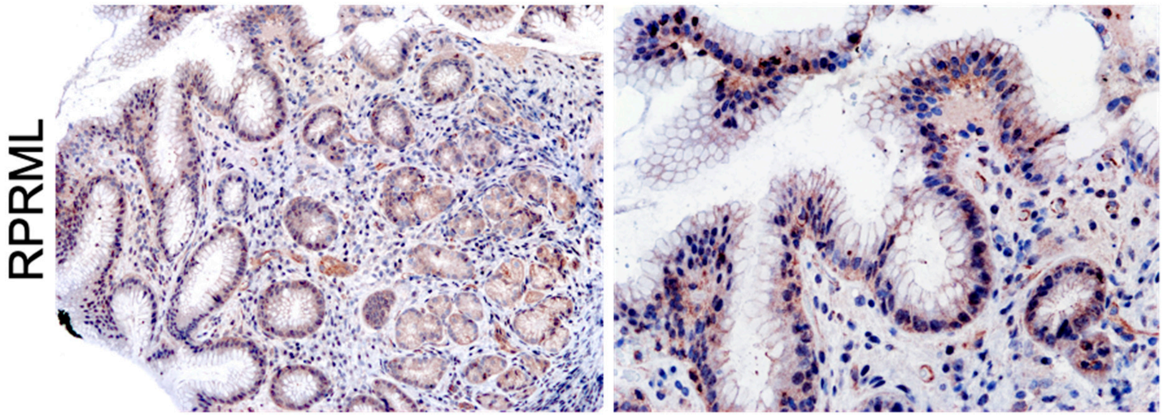

Supplementary Figure S1. RPRML expression in normal gastric mucosa. Representative image of RPRML IHC staining assay. Magnification: left panel, ×200; ×400, right panel.

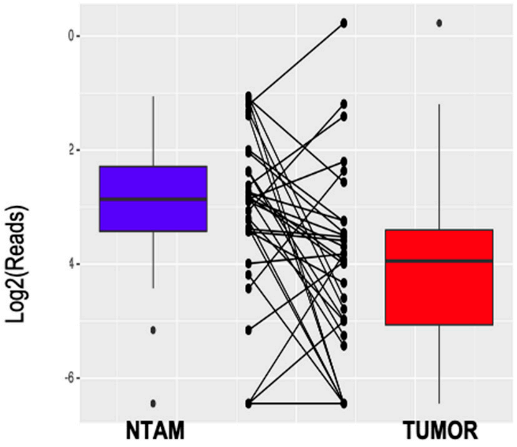

Supplementary Figure S2. *RPRML* transcript expression in the STAD dataset from TCGA repository. *RPRML* RNAseq levels in 32 matched pairs of tumor and NTAM. Shown are box plots (median ± IQR) and aligned dot plots of log2-normalized read counts per sample. Differential analysis was performed using the Wilcoxon signed-rank test (P = 0.0168).

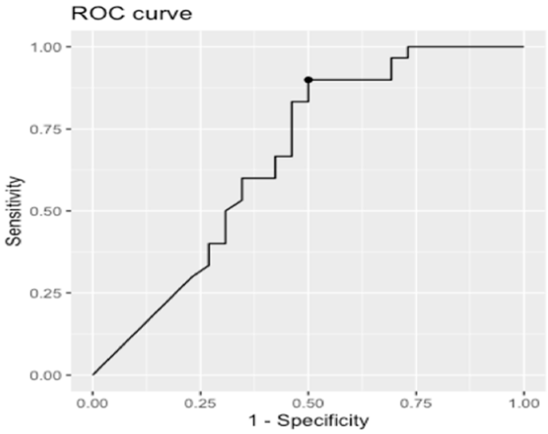

Supplementary Figure S3. ROC analysis for dividing patients into *RPRML* high- and low-expression groups. Outcome was defined as OS above the median of patients with advanced gastric cancer (25 months). For this outcome, the AUC was 0.665 and an optimal cut-off for RPRML IHC score of 0.162 was calculated by maximization of the Youden Index.

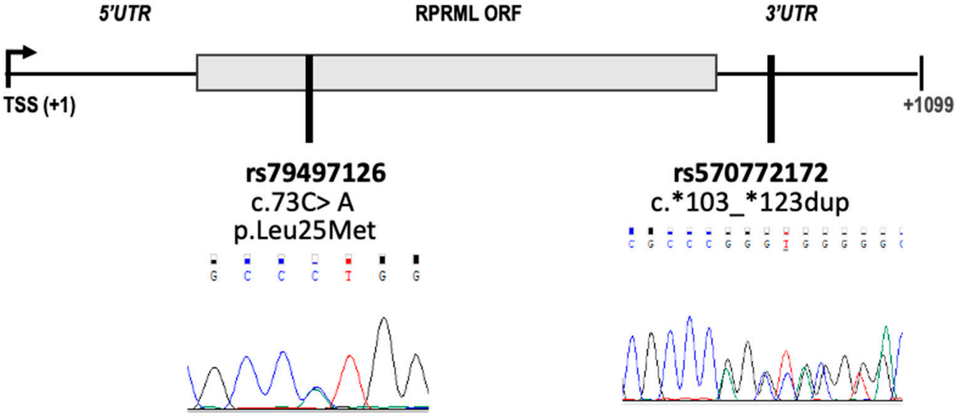

Supplementary Figure S4. Germline genetic screening of the *RPRML* gene in patients with familial gastric cancer. Two germline variants in the *RPRML* gene were found both previously reported in public databases (dbSNP and 1000 Genomes). Variant rs79497126 is a missense variant corresponding to the change from leucine to methionine at position 25. The effect of this mutation (p.L25M) was predicted using the PROVEAN tool (http://provean.jcvi.org/seq_submit.php). This change has a neutral effect, as both amino acids are apolar and aliphatic. The minor allele frequency was 0.120 in all populations from the 1000 Genomes database (n = 2504). Variant rs570772172 (c.*103_*123dup) corresponds to a 21–bp duplication in the *RPRML* 3'UTR. According to the guidelines for the interpretation of sequence variants, both germline variants identified in this familial gastric cancer cohort are classified as likely benign (Richards *et al. Genet Med*. 2015;17(5):405-24 doi 10.1038/gim.2015.30).

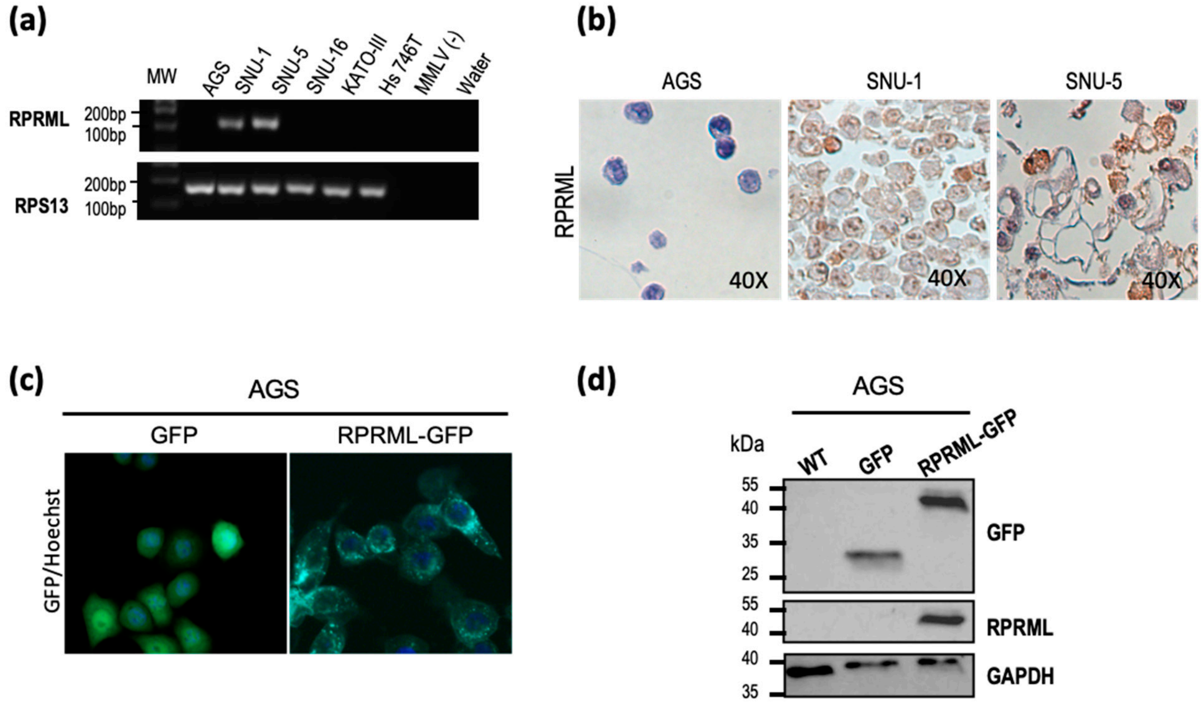

Supplementary Figure S5. (a) and (b) *RPRML* expression in GC cell lines. (**a**) Endogenous transcript expression of RPRML across six GC cell lines assessed by RT-PCR. MW: molecular weight DNA ladder; MMLV(-): reverse transcription negative control. **(b)** Endogenous protein expression of RPRML in AGS, SNU-1 and SNU-5 cell pellets assessed by immunocytochemistry. Only SNU-1 and SNU-5 cells express RPRML at mRNA and protein level. **(c) and (d)** *RPRML* overexpression in the AGS cell line. **(c)** Representative images of GFP- and RPRML-GFP–overexpressing AGS cells. Live cells were stained with Hoechst and analyzed by fluorescence microscopy at ×40 magnification. (**d**) Western blot analysis of WT, GFP-, and RPRML-GFP–overexpressing AGS cells using anti-GFP (GFP) or anti-RPRML antibodies. GAPDH was used as a loading control.

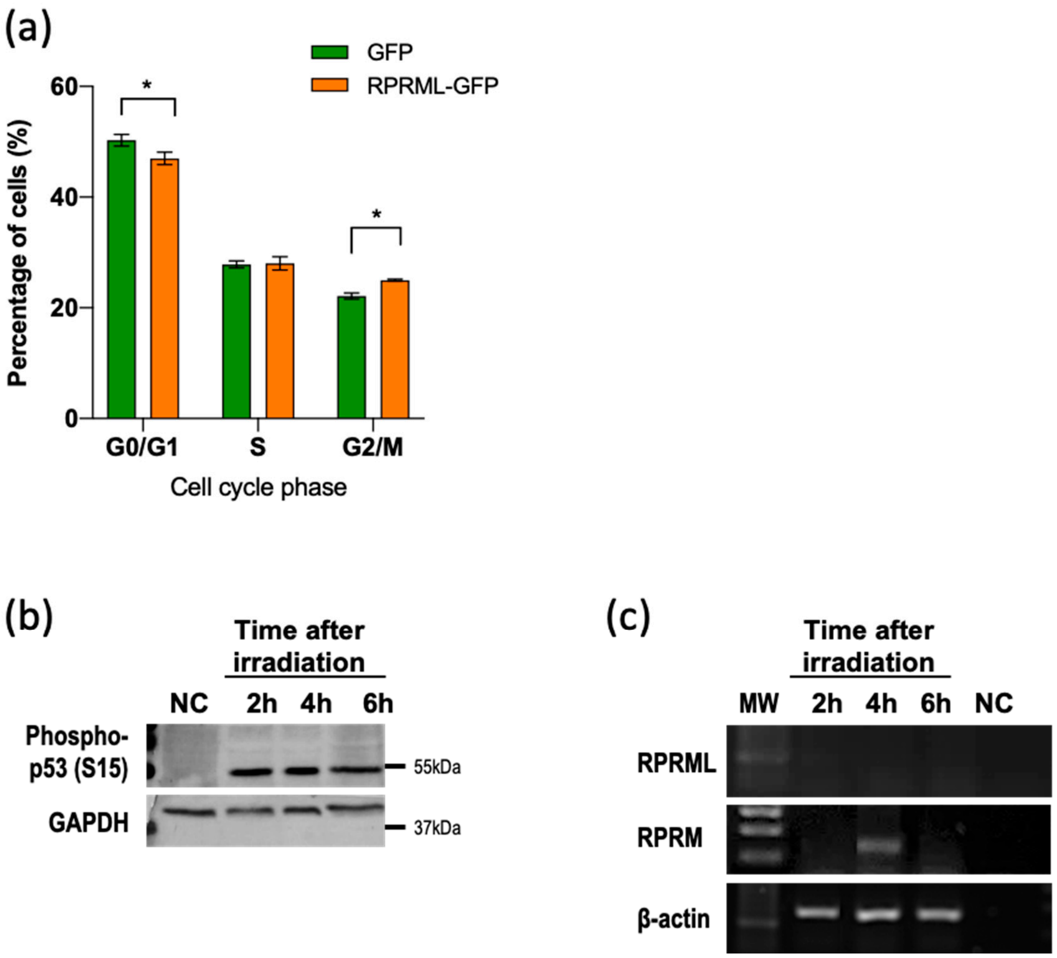

Supplementary Figure S6. (**a**) Effect of *RPRML* overexpression on cell cycle progression. DNA content was analyzed by flow cytometry of PI (propidium iodide)-stained cells. Data of 20,000 events/condition was recorded, and frequency histograms were analyzed using FCS Express DeNovo Software. Results represent the mean percentage of cells distributed in G0/G1, S, and G2/M from three independent experiments. Statistical analysis: Multiple *t*-test with the assumption of consistent SD for each phase (**P* < 0.05). **(b) and (c)** Effect of X-ray irradiation on *RPRML* expression. WT AGS cells were exposed to 12 grey X-ray. Total mRNA and protein lysates were extracted 2 h, 4 h, and 6 h post-irradiation. **(b)** DNA damage–induced activation of p53 was evaluated by western blot analysis of irradiated AGS cells using Phospho-p53 (Ser15) Antibody (Cell Signaling Technology, Cat# 9284, RRID:AB_331464). GAPDH was used as a loading control. Non-irradiated AGS cells were used as a negative control (NC). **(c)** *RPRML* and *RPRM* expression was evaluated by RT-PCR. β-Actin expression was used as a loading control. MW: molecular weight DNA ladder; NC: negative control (water).

Supplementary Table S1. RPRML IHC score according to clinicopathologic features from FORCE 1 clinical trial

Characteristic	N	%	RPRML IHC Score	
			Median	*P* Value
**Sex**				
Male	59	65.56	0.015	0.15[a]
Female	31	34.44	0.0375	
**Age (years)**				
≤50	13	14.61	0.013	0.83[c]
51-65	41	46.07	0.013	
>65	35	39.32	0.038	
**Lauren histological type**				
Intestinal	28	38.89	0.0375	0.56[c]
Diffuse	31	43.06	0.0313	
Mixed	13	18.06	0.0688	
**Signet-ring cell presence**				
No	57	63.33	0.0125	0.16[a]
Yes	33	36.67	0.0625	
**Localization**				
Proximal	18	20.93	0.0113	
Medial	37	43.02	0.0625	0.41[c]
Distal	27	31.4	0.0113	
Multiple	4	4.65	0.2875	
**Lymph node Metastasis**				
No	17	22.08	0.0625	0.18[b]
Yes	60	77.92	0.0144	
**Peritoneal involvement**				
No	88	97.78	0.0294	0.61[a]
Yes	2	2.22	0.0106	
**Hepatic Metastasis**				
No	85	97.7	0.0313	0.43[a]
Yes	2	2.3	0.2575	
**TNM Stage**				
I-II	32	35.56	0.0138	0.53[a]
III-IV	58	64.44	0.0343	

[a] *Wilcoxon sum rank test,* [b] *Welch's unequal variance t-test,* [c] *Kruskall-Wallis Test*

Supplementary Table S2. Analysis of RPRML expression according to other tissue markers from FORCE 1 clinical trial

**Tissue markers**	**N (%)**	**RPRML IHC Score**	
		**Median**	***P*-value**
**CISH-EBV**			
Negative	78 (86.7)	0.0406	0.21[b]
Positive	12 (13.3)	0.0012	
**PD-L1**			
Negative	64 (71.1)	0.0531	0.18[b]
Positive	26 (28.9)	0.0031	
**MSI**			
Negative	77 (85.6)	0.0375	0.41[a]
Positive	13 (14.4)	0.0138	
**p53**			
Negative	52 (57.8)	0.0169	0.88[a]
Positive	38 (42.2)	0.0375	
**p16**			
Lost	32 (36.8)	0.0025	0.38[b]
Normal	55 (63.2)	0.05	
**E-cadherin**			
Lost	15 (16.9)	0.01	0.66[a]
Normal	74 (83.1)	0.0344	
**Cleaved caspase 3**			
Low	20 (22.2)	0	0.043[b]
High	70 (77.8)	0.0562	

[a] *Wilcoxon sum rank test,* [b] *Welch's unequal variance t-test*

Supplementary Table S3. Univariate and multivariate associations between clinicopathologic variables and overall survival from the FORCE 1 clinical trial

		**Univariate Analysis**		**Multivariate Analysis**	
	**Mean (SD) or N (%)**	**Hazard Ratio**	***P*-value**	**Hazard Ratio**	***P*-value**
**Age**	62.5 (13.4)	1.02 (1.00-1.04)	0.122	1.01 (0.99-1.04)	0.165
**Gender**					
Male	59 (65.6)	Reference		Reference	
Female	31 (34.4)	1.34 (0.76-2.37)	0.313	1.15 (0.65-2.05)	0.631
**Stage**					
I-II	32 (35.6)	Reference		Reference	
III-IV	58 (64.4)	3.38 (1.69-6.78)	0.001	3.76 (1.86-7.62)	<0.001
**RPRML IHC-score**	0.1 (0.2)	0.30 (0.07-1.27)	0.102	0.17 (0.04-0.78)	0.022
